# Partial Body Mass Recovery After Caloric Restriction Abolishes Improved Glucose Tolerance in Obese, Insulin Resistant Rats

**DOI:** 10.3389/fendo.2020.00363

**Published:** 2020-06-10

**Authors:** Manuel A. Cornejo, Julie Nguyen, Joshua Cazares, Benny Escobedo, Akira Nishiyama, Daisuke Nakano, Rudy M. Ortiz

**Affiliations:** ^1^School of Natural Sciences, University of California, Merced, Merced, CA, United States; ^2^Department of Pharmacology, Faculty of Medicine, Kagawa University, Kagawa, Japan

**Keywords:** caloric restriction, gluconeogenesis, adipokines, insulin resistance, lipolysis

## Abstract

Caloric restriction, among other behavioral interventions, has demonstrated benefits on improving glycemic control in obesity-associated diabetic subjects. However, an acute and severe intervention without proper maintenance could reverse the initial benefits, with additional metabolic derangements. To assess the effects of an acute caloric restriction in a metabolic syndrome model, a cohort of 15-week old Long Evans Tokushima Otsuka (LETO) and Otsuka Long Evans Tokushima Fatty (OLETF) rats were calorie restricted (CR: 50% × 10 days) with or without a 10-day body mass (BM) recovery period, along with their respective *ad libitum* controls. An oral glucose tolerance test (oGTT) was performed after CR and BM recovery. Both strains had higher rates of mass gain during recovery vs. *ad lib* controls; however, the regain was partial (ca. 50% of *ad lib* controls) over the measurement period. Retroperitoneal and epididymal adipose masses decreased 30% (8.8 g, *P* < 0.001) in OLETF; however, this loss only accounted for 11.5% of the total BM loss. CR decreased blood glucose AUC 16% in LETO and 19% in OLETF, without significant decreases in insulin. Following CR, hepatic expression of the gluconeogenic enzyme, PEPCK, was reduced 55% in OLETF compared to LETO, and plasma triglycerides (TG) decreased 86%. Acute CR induced improvements in glucose tolerance and TG suggestive of improvements in metabolism; however, partial recovery of BM following CR abolished the improvement in glucose tolerance. The present study highlights the importance of proper maintenance of BM after CR as only partial recovery of the lost BM reversed benefits of the initial mass loss.

## Introduction

Obesity and its associated metabolic disorders are significant health problems that have sustained global attention and concern for over the past 3 decades, with an alarming, increasing trend (1, 2). Unfortunately, obesity is related to 300,000 deaths per year in the United States alone (3), most of them attributed to class II/III obesity (3.8% excess deaths for females, 2.5% males compared to a cohort with normal BMI) (4) and type 2 diabetes mellitus (T2DM) was the underlying cause of death of 75,486 adults in the U.S. in 2013 (5). The adult, obese U.S. population increased from 30.5% in 1999 to 39.6% in 2016 (1). Excess body mass (BM) and obesity increase morbidity and mortality associated with numerous complications, including T2DM, dyslipidemia, hypertension, and atherosclerosis (6, 7). Behavioral interventions such as a low-carbohydrate diet (8), caloric restriction (CR) (9), vigorous physical activity (PA), or some combination (10) have demonstrated benefits for improving glycemic control and adiposity in obesity-associated diabetic subjects. However, rapid loss of BM can be associated with adipose mass regain and an increase of HOMA-IR over time if vigorous PA or CR is not maintained (11). The detriments (increased adiposity, insulin resistance, adipokines, and triglycerides) observed following the regain of the lost BM is known as the “rebound effect” (11, 12). Furthermore, CR alone may be associated with increased loss of lean tissue (fat-free mass) and the associated water vs. adipose mass loss (70% of total mass loss derived from water and 5% from lean tissue, vs. 25% from adipose) (13), especially during prolonged semi-starvation conditions where lean tissue loss can account for up to 41% of the BM loss (14), which may minimize greater potential benefits. However, the metabolic adjustments associated with CR during metabolic syndrome and following a subsequent regain in BM are not well-elucidated.

Caloric restriction leads to glycogen depletion in muscle and liver, leading to increased lipolysis and formation of ketone bodies, while decreasing glucose output via inhibition of gluconeogenesis and glycogenolysis (15). However, increased utilization of lipids as a consequence of CR can lead to insulin resistance (IR) (16).

Adipose tissue secretes many biologically active proteins including leptin and adiponectin (17). Decreased leptin during adipose mass loss can contribute to increased hunger, lower metabolic rate and mass regain (18). Conversely, increased leptin decreases insulin sensitivity, contributing to systemic hyperinsulinemia and T2DM (19). Adiponectin has protective effects against cardiovascular disease, is negatively correlated to triglyceride levels, is positively correlated to HDL levels (20), and enhances insulin action when administered to animals during conditions of increased fat oxidation (21).

The goal of this study was to assess the benefits of acute CR on glycemic control and lipid metabolism in a model of metabolic syndrome and comparing these effects to those induced by subsequent regain in BM in the form of adipose tissue, or “rebound effect.” Previous studies found an increase in fatty acid synthesis and liver lipid accumulation to be principal consequences of the rebound effect after a moderate (30%) CR in OLETF (22), and increased leptin and peripheral glucose resistance after fat mass recovery following a 40–50% CR in obesity-prone Wistar rats (23). However, other aspects of this phenomenon are yet to be elucidated such as humoral factors driving the change in glucose tolerance, adiposity, and arterial pressure.

The OLETF model resembles the pathological features of human metabolic syndrome including late onset hyperglycemia, mild obesity (24, 25), insulin resistance, hyperlipidemia, and hypertension (26–28). We hypothesized that: (1) CR will improve systemic insulin sensitivity and adipokine profile while decreasing hepatic gluconeogenesis and increasing lipolysis and NEFA uptake, and (2) mass recovery will reverse the improvements realized by CR-induced BM.

## Materials and Methods

All experimental procedures were reviewed and approved by the institutional animal care and use committee of Kagawa Medical University (Kagawa, Japan).

### Animals

**Figure 1:**
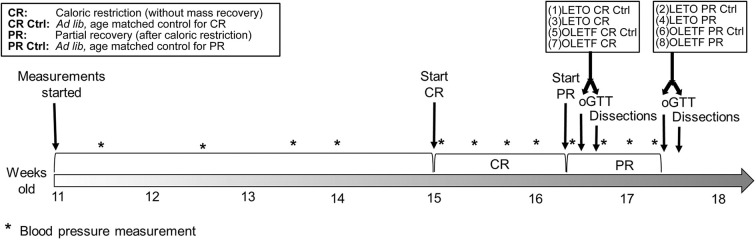
Study timeline. For both strains, first dissected groups were CR and CR control, and last dissected groups were PR and PR control.

Male, lean strain-control, Long Evans Tokushima Otsuka (LETO) (*n* = 29) and obese, insulin resistant Otsuka Long Evans Tokushima Fatty (OLETF) (*n* = 29) rats (Otsuka Pharmaceutical Co., Ltd., Tokushima, Japan) of 11 weeks of age were fed *ad libitum* with standard laboratory rat chow (MF; Oriental Yeast Corp., Tokyo, Japan) for 4 weeks. At 15 weeks, rats were randomly assigned to one of the following groups: (1) LETO *ad libitum* control for CR (*n* = 8), (2) LETO *ad libitum* control for partial recovery of BM (*n* = 7), (3) LETO with 50% caloric restriction (LETO CR) (*n* = 7), (4) LETO with 50% CR followed by *ad libitum* feeding, resulting in partial recovery of BM (LETO PR; 73% recovery of mass loss) (*n* = 7), (5) OLETF *ad libitum* control for CR (*n* = 7), (6) OLETF *ad libitum* control for PR (*n* = 8), (7) OLETF 50% CR (*n* = 7), and (8) OLETF PR (*n* = 7; 59% recovery of mass loss) (Figure 1). Total mass recovery was not achieved purposefully to best assess the impacts of partial recovery. The LETO strain was restricted as well to be able to discriminate between physiological CR- driven changes and the changes related to metabolic syndrome, as well as to contrast with the baseline levels in the OLETF animals. All animals were maintained in groups of two animals per cage at the start of the study to minimize stress (29) and one per cage during the CR phase. *Ad libitum* food intake per rat was calculated as the mean intake for double occupancy cages. Animals were maintained in a specific pathogen-free facility under controlled temperature (23°C) and humidity (55%) with a 12-h light, 12-h dark cycle. All animals were given free access to water for the entire study.

### Blood Pressure

Systolic blood pressure (SBP) was consistently measured in triplicate in conscious rats by tail-cuff plethysmography (BP-98A; Softron Co., Tokyo, Japan) (*n* = 6/group) as previously described (26, 30, 31). Rats were acclimated to the tube restraints prior to measurements. Measurements were taken after feeding once a week during the first 4 weeks of the study (LETO vs. OLETF before start of CR), every other day until 16 weeks for CR vs. control and 17 weeks for PR vs. control groups. Repeated measures with a percent coefficient of variability (CV) >15% were excluded.

### Body Mass (BM) and Food Intake

BM and food intake were measured daily to calculate the appropriate amount of chow to be given to CR and PR groups. At 15 weeks, all groups except LETO and OLETF *ad lib* controls were given 50% of mean food intake of control group for the next 10 days resulting in mean decreases in BM of 12 and 14% for LETO and OLETF, respectively. Immediately following this 10-day CR phase, a subset of the remaining animals (*n* = 7–8) (from the CR cohorts) were fed *ad libitum* again for 1 week, representing the PR phase.

### Oral Glucose Tolerance Test (oGTT)

After the 10 days of CR, oGTTs were performed in half of the *ad lib* control groups (LETO and OLETF, *n* = 7/group) and in all the CR groups (LETO and OLETF, *n* = 7/group). oGTTs were performed in the remaining animals representing the PR groups 7 days later. oGTTs were performed as previously detailed in our hands (26). Briefly, a 2 g/kg glucose bolus was given by gavage to overnight-fasted (14+ h) rats. Blood was collected via the caudal vein before gavage and 15, 30, 60, and 120 min after.

### Dissections

Three days after oGTTs, animals were fasted overnight and tissues collected the subsequent morning. After BM measurements were obtained, animals were anesthetized with 100 mg/kg i.p. pentobarbital injection and arterial blood was collected via the abdominal aorta into chilled vials containing a cocktail of 50 mmol/L EDTA, 5000 KIU aprotinin, and 0.1 mmol sitagliptin phosphate (DPP4 inhibitor). Vials were kept on ice until they could be centrifuged. Systemic perfusion with chilled PBS was performed via the same artery with an incision in the inferior vena cava as the exit point and proceeded until no blood was present. Thereafter, organs and fat depots were rapidly removed, weighed, and snap frozen in liquid nitrogen. Frozen samples were kept at −80°C until analyzed. Blood samples were centrifuged (3,000 g, 15 min at 4°C), and the plasma was transferred to cryo-vials and immediately stored at −80°C.

### Biochemical Analyses

Plasma triglycerides (TG) was measured using a Hitachi 7020 chemistry analyzer (Diamond Diagnostics, Massachusetts, USA), and total protein content was measured by the Bradford assay (Bio-Rad Laboratories, CA, USA). Plasma and liver non-esterified fatty acids (NEFA), and liver diacylglycerol (DAG) were measured using commercially available kits (Wako, Osaka, Japan; MyBioSource, San Diego, USA). Hepatic NEFA and DAG measurements were performed following whole lipid extraction from an aliquot of liver (20–30 mg) by the method described previously (32) and later modified (18). Lipase activity was measured in plasma as previously described (33). Plasma insulin (Wako, Osaka, Japan), total GLP-1 (Millipore, Burlington, USA), plasma aldosterone, and serum leptin and adiponectin (R&D Systems, Minneapolis, USA) were measured using commercially available ELISA kits (34, 35). All samples were analyzed in duplicate and run in a single assay with intra-assay and percent CV of <10% for all assays. Amino acids were measured by GCTOF MS (West Coast Metabolomic Center, University of California Davis, CA, USA). Creatinine and urea were measured by colorimetric method (QuantiChrom, Bioassay Systems, CA, USA) to better assess changes in lean mass [i.e., catabolism; (8)]. Serum electrolytes were measured by ISE (EasyLyte, MA, USA).

### Protein Expression by Western Blot

Frozen kidney cortex was homogenized in 200 μl RIPA buffer (Thermo Fisher Scientific, NY, USA) and frozen liver was homogenized in either 200 μl RIPA (for G6Pc) or 150 μl STM buffer (250 mM sucrose, 50 mM Tris–HCl pH 7.4, and 5 mM MgCl_2_) containing 1% protease and 3% phosphatase inhibitor cocktail (Sigma-Aldrich, St. Louis, USA). Tissue homogenate was then sonicated for 20 s, centrifuged (15,120 g × 15 min for kidney and 800 g × 15 min for liver), and the supernatant total protein content was measured by the Bradford assay (Bio-Rad Laboratories, Hercules, CA).

Predetermined amounts of total protein (TP) for kidney (40 μg) and liver homogenate (20 μg) were resolved in a 10% Tris-HCl SDS gel. Proteins were electroblotted using the Bio-Rad Trans Blot onto a 0.45 μm Inmovilon-FL polyvinylidene difluoride (PVDF) membrane for 2 h at 100 V. Membranes were blocked with 25% Odissey Blocking Buffer (PBS) and incubated 16 h at 4°C with primary antibody against SGLT2 (1:200 dilution), PEPCK-C (1:200 dilution), G6Pc (1:500 dilution) (Santa Cruz Biotechnology, Dallas, USA). Membranes were washed with TBS 1% Tween-20 and incubated for 1 h with a secondary antibody (LI-COR Biosciences, Lincoln, USA) diluted 1:10,000, rewashed and visualized using a Li-Cor Odissey Imaging System. Densitometry values were quantified by ImageJ software (NIH) and further normalized by correcting for densitometry values of a representative protein band stained with Ponceau S. Results are reported as percentage of expression compared to LETO baseline (CR control) unless stated otherwise.

### Statistics

Means (±SE) were compared by two-way ANOVA for strain x treatment and interaction, with the Holm-Sidak method for *post-hoc* multiple comparison after excluding outliers by extreme studentized deviate test with α = 0.05. Level of significance is considered for age-paired groups only, except for CR vs. PR. Glucose tolerance was assessed by comparing mean AUC values obtained from the glucose profiles during the oGTT. The AUC values were also compared by two-way ANOVA. SBP measurements were compared per day by one-way (before CR) or two-way (during CR and PR) ANOVA. Mass increment and food intake per day were also compared by one-way ANOVA before intervention and two-way ANOVA during CR and PR. Repeated measures ANOVA was performed in glucose and insulin data, but avoided for SBP analysis as samples were randomized and measures with >15% CV were excluded. Relationships between dependent and independent variables were evaluated by simple regression (except for insulin and DAG analysis, where a 4th order regression and power regression were used, respectively). Correlations were evaluated using Pearson correlation coefficients. Means, regression, and correlations were considered significantly different at *P* < 0.05. Statistical analyses were performed with SigmaPlot 12.5 software (Systat Software Inc., San Jose, CA).

## Results

### Increased *ad lib* Refeeding After CR Increases the Rate of Mass Gain

**Figure 2:**
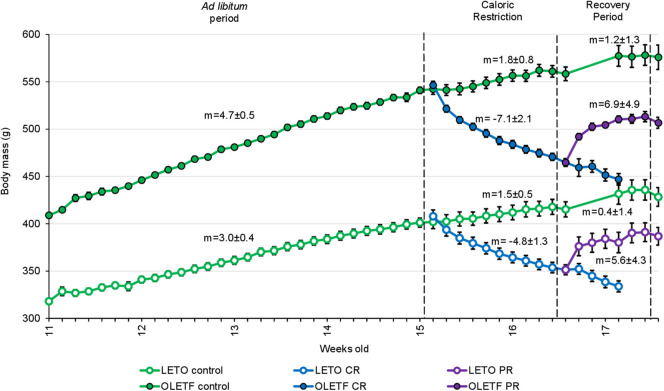
Mean ± SE animal body mass per week of age (*n* = 7, except for Controls before recovery period, where *n* = 14). m, mean slope of the line ± SE (body mass increase in g/day).

Slope analysis was performed to better appreciate the impacts of the treatments (CR and PR) on the changes in BM (Figure 2). Mean mass increment per day decreased in LETO *ad lib* control from 3.0 ± 0.4.g/day (*r*^2^ = 1) before CR to 0.4 ± 1.4 g/day (*r*^2^ = 0.63; *P* < 0.001) during recovery period, and decreased from 4.7 ± 0.5 (*r*^2^ = 1) to 1.2 ± 1.3 g/day (*r*^2^ = 0.55; *P* < 0.001) in OLETF. Mean mass decrease during CR was −4.8 ± 1.3 g/day (*r*^2^ = 0.96) and −7.1 ± 2.1 g/day (*r*^2^ = 0.93) for LETO and OLETF, respectively (*P* = 0.392 LETO vs. OLETF). During partial recovery, mean mass increase was 5.6 ± 4.3 g/day (*r*^2^ = 0.73) for LETO and 6.9 ± 4.9 g/day (*r*^2^ = 0.75) for OLETF (*P* = 0.847 LETO vs. OLETF). During the regain phase, animals in both strains regained ~50% of the lost BM in 7 days, and the rates of regain were not different between the two strains, despite *ad lib* food intake increase in LETO during PR, compared to before CR (19.6 ± 0.6 vs. 23.7 ± 1.1 g/day; *P* = 0.004 in LETO and 24.4 ± 1.8 vs. 29.0 ± 2.0 g/day; *P* = 0.100 in OLETF).

### CR Decreased Fat Depots, but Did Not Increase With Partial Recovery

**Figure 3:**
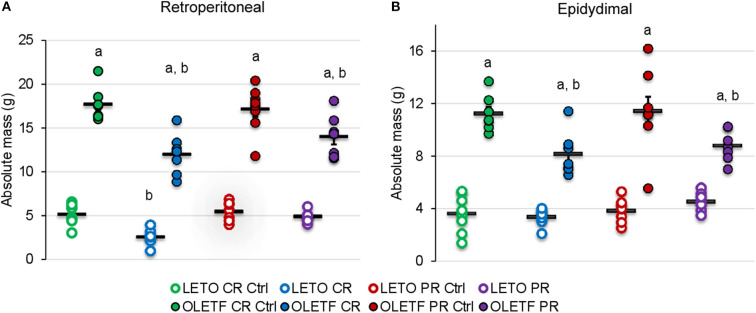
Mean ± SE absolute (g) **(A)** retroperitoneal and **(B)** epididymal adipose depots. ^a^*P* < 0.05 vs. LETO. ^b^*P* < 0.05 vs. Control.

The principal intraperitoneal fat depots were measured to better ascertain the degree to which the treatments (CR and PR) altered adiposity. Mean retroperitoneal adipose mass decreased after CR in both strains; by half in LETO (*P* = 0.037) and by 32% in OLETF (*P* < 0.001; Figure 3A). Retroperitoneal mass did not significantly increase after PR in OLETF (17% above CR; *P* = 0.121), but recovered to basal levels in LETO (10% below CR Control; *P* = 0.841; Figure 3A). In LETO, mean epididymal fat mass remained unchanged after CR (*P* = 0.999) and did not significantly increase with PR (25%: *P* = 0.751). Conversely, in OLETF, epididymal fat decreased 27% (*P* = 0.009) after CR and remained reduced after PR (23% below PR Control; *P* = 0.018; Figure 3B). Similar changes were observed after calculating the relative masses (Table 1). There were no significant changes in the relative masses of heart, kidney or liver in any of the strains following CR (Table 1). However, there was an increase in plasma amino acids independent of decreased protein intake during CR suggesting that lean tissue catabolism was primarily from skeletal muscle (Table 2).

**Table 1:** Mean ± SE relative (%) body composition at endpoint.

**Strain**	**Treatment**	**Retro (%)**	**Epi (%)**	**Heart (%)**	**Liver (%)**	**Kidney (%)**	**Other (%)**
LETO	CR control	1.3 ± 0.1	0.9 ± 0.1	0.4 ± 0	2.8 ± 0.1	1.5 ± 0.1	93.0 ± 0.3
	CR	0.8 ± 0.1	1.0 ± 0.1	0.3 ± 0	2.5 ± 0.1	1.4 ± 0.0	94.1 ± 0.1
	PR control	1.3 ± 0.1	0.9 ± 0.1	0.3 ± 0	2.4 ± 0.2	1.1 ± 0.0	93.9 ± 0.3
	PR	1.3 ± 0.1	1.2 ± 0.1	0.3 ± 0	2.7 ± 0.1	1.3 ± 0.0	93.3 ± 0.1
OLETF	CR control	3.4 ± 0.1^a^	2.2 ± 0.1^a^	0.3 ± 0	2.8 ± 0.1	1.3 ± 0.0	89.9 ± 0.3^a^
	CR	2.7 ± 0.2^a^^,^^b^	1.8 ± 0.1^a^	0.3 ± 0	2.7 ± 0.2	1.4 ± 0.0	91.1 ± 0.3^a^^,^^b^
	PR control	3.2 ± 0.1^a^	2.1 ± 0.2^a^	0.3 ± 0	2.8 ± 0.1	1.4 ± 0.1	90.2 ± 0.4^a^
	PR	2.8 ± 0.2^a^	1.8 ± 0.1^a^	0.3 ± 0	2.8 ± 0.1	1.2 ± 0.0	91.1 ± 0.2^a^

*Retro, retroperitoneal fat depot; Epi, epididymal fat depot; other, accounts for the rest of the body mass*.

a *P < 0.05 vs. LETO*.

b *P < 0.05 vs. Control*.

Table 2Mean ± SE plasma biochemical markers and electrolytes concentrations.

**Table:** 

**Strain**	**Treatment**	**Urea** **(mmol/L)**	**Creatinine** **(μmol/L)**	**SI urea: creatinine ratio**	**Plasma protein** ** (μmol/L)**	**Plasma amino acids** ** (% vs. LETO CR Ctrl)**
LETO	CR control	9.0 ± 0.6	28.8 ± 2.9	357 ± 51	625 ± 32	100 ± 3
	CR	11.0 ± 0.5	50.3 ± 5.1^b^	245 ± 19	602 ± 28	118 ± 9
	PR Control	9.8 ± 2.0^b^	49.6 ± 2.1^b^	244 ± 24	706 ± 25	126 ± 4
	PR	12.7 ± 0.2	41.8 ± 3.8	290 ± 19	674 ± 30	96 ± 4
OLETF	CR Control	9.6 ± 0.4	32.5 ± 2.9	312 ± 44	727 ± 47^a^	91 ± 3
	CR	5.2 ± 1.0^a^^,^^b^	31.9 ± 5.0^a^	159 ± 22^b^	655 ± 35	130 ± 14^b^
	PR Control	9.9 ± 0.6^c^	42.6 ± 2.0	243 ± 21	847 ± 41^a^	96 ± 5^c^
	PR	10.0 ± 0.4^c^	40.5 ± 1.8	245 ± 13	765 ± 29^c^	92 ± 5^c^

**Table:** 

**Strain**	**Treatment**	**Aldosterone** **(pmol/L)**	**Cl**^**−**^ **(mmol/L)**	**K**^**+**^ **(mmol/L)**	**Na**^**+**^ **(mmol/L)**
LETO	CR Control	4.8 ± 0.9	101 ± 2	7.4 ± 1.1	140 ± 2
	CR	7.4 ± 1.5	98 ± 1	5.7 ± 0.1	141 ± 1
	PR Control	7.4 ± 1.2	102 ± 2	6.1 ± 0.3	142 ± 2
	PR	15.2 ± 1.7	93 ± 4	6.8 ± 0.2	130 ± 5
OLETF	CR Control	10.4 ± 2.6	98 ± 2	5.8 ± 0.2	138 ± 3
	CR	5.0 ± 2.5	101 ± 0	5.5 ± 0.3	141 ± 1
	PR Control	10.1 ± 0.7	100 ± 1	7.9 ± 0.2^a^,^c^	139 ± 1
	PR	9.1 ± 2.3^b^^,^^c^	101 ± 1	7.1 ± 0.2	140 ± 1

a *P < 0.05 vs. LETO*.

b *P < 0.05 vs. Control*.

c *P < 0.05 vs. CR*.

### CR Did Not Ameliorate the Increase in SBP Associated With the Metabolic Syndrome in OLETF

**Figure 4:**
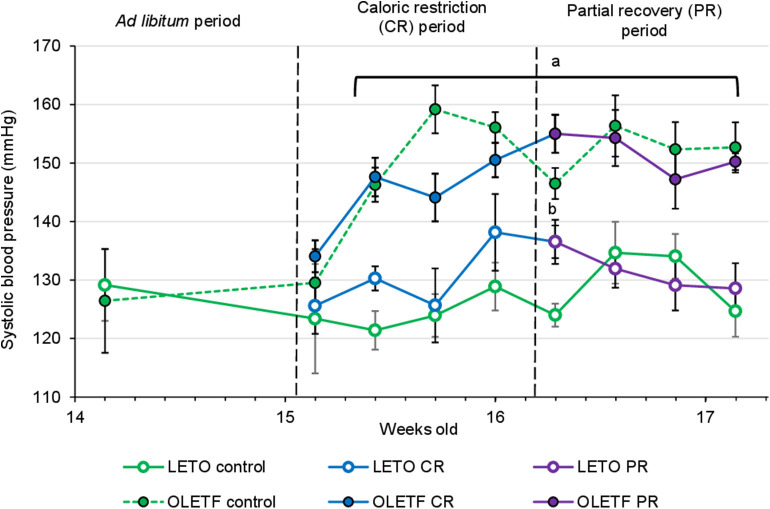
Mean ± SE values of systolic blood pressure (SBP) by weeks of age (*n* = 6). Solid lines represents CR and Recovery groups, whereas dashed lines represents Control groups. ^a^*P* < 0.05 vs. LETO. ^b^*P* < 0.05 vs. Control.

Mean SBP for both LETO and OLETF was similar before CR (129 ± 4 vs. 126 ± 4 mmHg; *P* = 0.631, respectively). SBP increased in OLETF compared to LETO 2 days after starting CR (121 ± 2 vs. 146 ± 3 mmHg; *P* < 0.001), and this difference was maintained through the rest of the study. However, there was not a significant difference between control groups and CR or PR in either of the strains, with the exception for LETO Control vs. CR at the end of the CR period (124 ± 4 vs. 137 ± 3 mmHg; *P* = 0.026, respectively; Figure 4). Serum Na^+^ and Cl^−^ were unaltered between strains or after CR; however, K^+^ was 30% higher (*P* = 0.045) in OLETF PR control (7.9 ± 0.2 mmol/L) compared to LETO (6.1 ± 0.3 mmol/L). Basal serum aldosterone was 2-fold higher (*P* = 0.024) in OLETF compared to LETO (10.4 ± 2.6 vs. 4.8 ± 0.9 mmol/L × 10^−9^). However, CR did not change aldosterone concentration in any of the strains.

### CR Improves Glucose Tolerance and Insulin Resistance, but PR Completely Negates These Improvements

**Figure 5:**
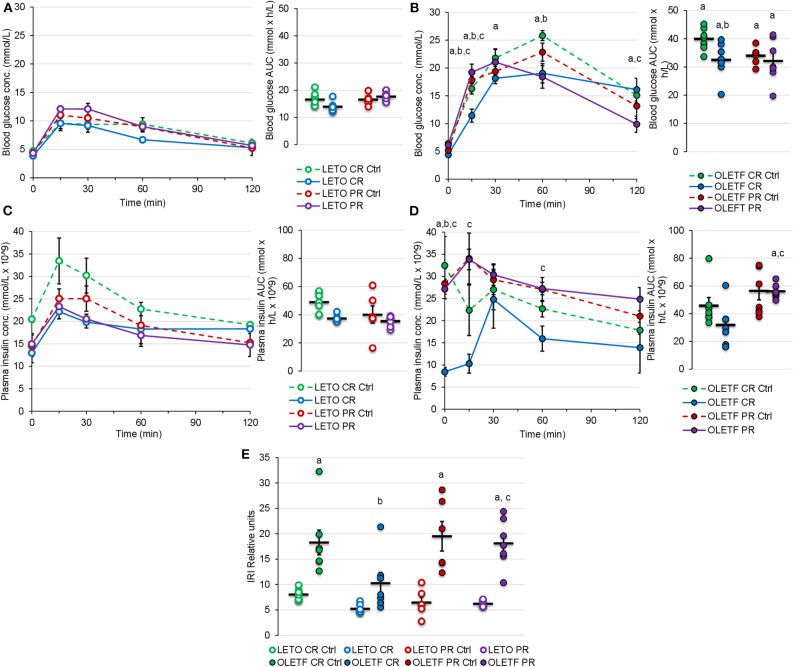
Mean ± SE blood glucose concentration (mmol/L) respect to time (min) and AUC (mmol × h/L) during oGTT for **(A)** LETO and **(B)** OLETF. Mean ± SE plasma insulin concentration (mmol/L × 10^−9^) with respect to time (min) and AUC calculations (mmol × h/L × 10^−9^) during oGTT for **(C)** LETO and **(D)** OLETF. Mean ± SE **(E)** insulin resistance index (IRI). ^a^*P* < 0.05 vs. LETO. ^b^*P* < 0.05 vs. Control. ^c^*P* < 0.05 vs. CR.

Glucose tolerance tests with corresponding insulin measurements and subsequent insulin resistance index (IRI) calculations were performed to quantify the functional metabolic effects of the treatment-induced alterations in BM (IRI = Glucose AUC × Insulin AUC/100). Mean blood glucose AUC was 2.4-fold higher in OLETF compared to LETO at baseline (*P* < 0.001). OLETF had a more pronounced decrease in glucose (19%, *P* = 0.010) compared to a non-significant decrease in LETO (16%, *P* = 0.758) after CR. However, LETO mean AUC increased 7% above control (*P* = 0.948), while OLETF maintained 5% below control after PR (*P* = 0.827), with a minimal 1% reduction vs. CR (*P* = 0.882). Blood glucose concentration peaked at 60 min for OLETF and 15 min for LETO after CR, and peaked at 30 min in OLETF after the recovery period (Figures 5A,B).

Mean plasma insulin AUC was 8% higher in LETO at 16 weeks, but 35% higher (*P* = 0.035) in OLETF at 17 weeks. Mean plasma insulin in LETO was lower, and higher in OLETF, for PR Ctrl compared to CR control, however this differences were non-significant and could be attributed to intra-subject variability rather than age effect. Plasma insulin AUC had a non-significant decrease in both strains after CR (25% in LETO and 31% in OLETF). Plasma insulin concentration peaked at 15 min for all groups except for OLETF CR, which peaked at 30 min (Figures 5C,D).

Mean baseline IRI was 128% higher in OLETF compared to LETO (*P* < 0.001), and decreased 44% (*P* = 0.015) in OLETF after CR. However, IRI returned to baseline levels after PR (Figure 5E). It is important to consider that glucose AUC influenced the IRI calculations more profoundly than the insulin AUC, suggesting that impaired glucose handling/metabolism (most likely at the cellular level) is the primary factor contributing to the metabolic derangement associated with the condition as opposed to impaired glucose-stimulated insulin secretion (i.e., insulin response) in untreated OLETF.

### Basal Expression of Gluconeogenic Enzymes Is Higher in OLETF

**Figure 6:**
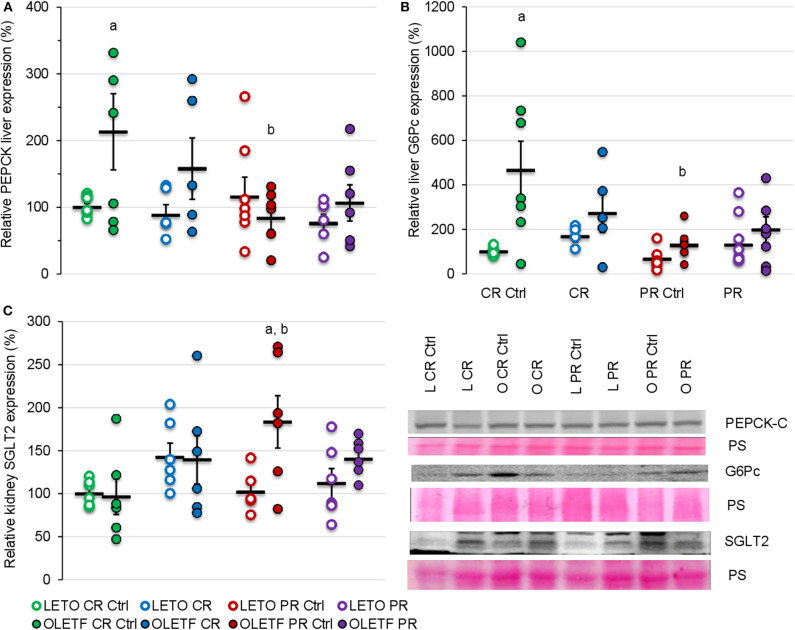
Mean ± SE relative expression of **(A)** liver PEPCK, **(B)** liver G6Pc, and **(C)** kidney SGLT2 expression. ^a^*P* < 0.05 vs. LETO. ^b^*P* < 0.05 vs. Control.

To further help elucidate potential mechanisms that contribute to the changes in glucose tolerance induced by the treatments, the protein expressions of hepatic PEPCK and G6Pc were quantified as markers of hepatic gluconeogenesis. Decrease in expression of these enzymes after CR was not significant; however, basal expression of cytosolic phosphoenolpyruvate carboxykinase (PEPCK-C) in OLETF was 113% higher than LETO (*P* = 0.007; Figure 6A) and mean basal expression of the downstream gluconeogenic enzyme, glucose-6-phosphatase (G6Pc) was 4.6-fold higher (*P* < 0.001) in OLETF control compared to LETO control (Figure 6B). CR and PR had no detectable effects on protein expressions of either enzyme.

### Kidney SGLT2 Expression Increases in OLETF With Partial Recovery of BM

Given the benefits of CR on glucose tolerance and IRI, kidney SGLT2 was measured to assess the potential of its contribution to the improvements in glucose metabolism. CR did not significantly change the mean relative expression of SGLT2 in either strain (Figure 6C). Regardless of the treatment, the expression remained relatively constant in LETO. However, there was a significant increase in OLETF PR control compared to OLETF CR control (183 vs. 97%; *P* = 0.015) and LETO PR control (98%; *P* = 0.006), which likely reflects the progression of the metabolic syndrome in this strain (Figure 6C).

### The CR-Induced Decrease in Adipose in OLETF Is Associated With a Concomitant Decrease in Serum Leptin and Increased Adiponectin After PR

**Figure 7:**
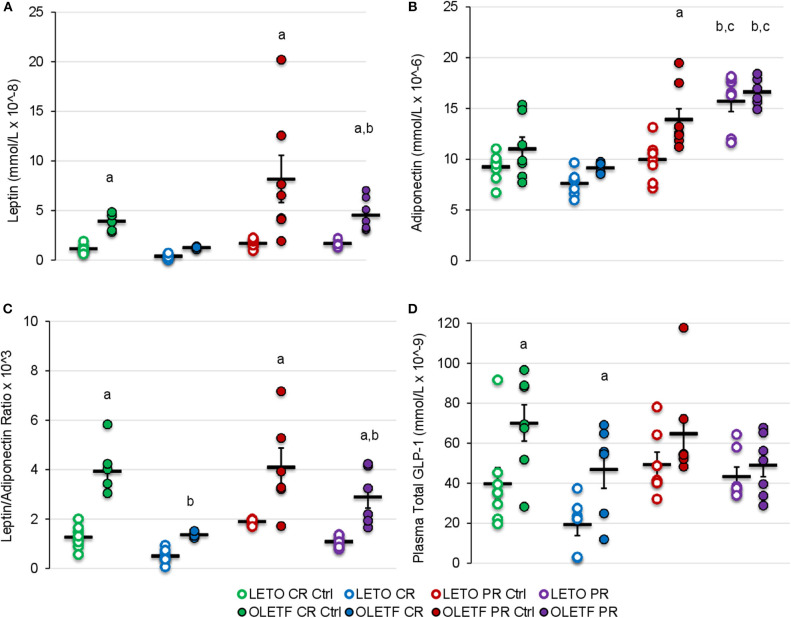
Mean ± SE **(A)** serum leptin (mmol/L × 10^−8^), **(B)** adiponectin (mmol/L × 10^−6^), **(C)** leptin/adiponectin ratio × 10^3^, and **(D)** plasma total GLP-1 (mmol/L × 10^−9^). ^a^*P* < 0.05 vs. LETO. ^b^*P* < 0.05 vs. Control. ^c^*P* < 0.05 vs. CR.

Given the changes in the principal i.p. adipose depots with the treatments, the adipose-derived hormones, leptin and adiponectin, were measured to gain insights on the sensitivity of adipose to the treatment's effects and their potential to contribute to the metabolic effects observed. Mean serum leptin was higher in OLETF control (*P* = 0.038) than LETO control, representative of the increased adiposity associated with the model, and decreased 3.1-fold after CR without reaching significance (CR Control: 3.9 ± 0.3 vs. CR: 1.3 ± 0.1 mmol/L; *P* = 0.177). However, in OLETF mean serum leptin was maintained below control after partial recovery (PR Control: 8.2 ± 2.4 vs. PR: 4.5 ± 0.6 mmol/L; *P* = 0.028; Figure 7A). Regardless of treatment, plasma leptin remained constant in LETO throughout the study. Serum adiponectin remained unchanged in both strains at baseline and after CR, but increased 61% (*P* < 0.001) after PR in LETO and 18% (*P* = 0.05) in OLETF (Figure 7B). Baseline leptin: adiponectin ratio was 3-fold higher in OLETF vs. LETO; however, the ratio decreased 2-fold in LETO and 3-fold in OLETF (*P* < 0.001) after CR (Figure 7C).

### Strain-Effect on Plasma GLP-1 Remains Despite CR

Mean plasma GLP-1 concentrations were greater in OLETF compared to LETO at baseline and following CR (*P* < 0.05), despite substantial, however non-significant decreases in both strains with CR (Figure 7D). Significant differences in mean GLP-1 concentrations following PR were not detected. The reduction of circulating GLP-1 in OLETF after CR and the greater basal levers in OLETF compared to LETO suggests that, although GLP-1 improves glucose tolerance by augmenting peripheral insulin action (16, 36), it may not be a key factor in improving glucose tolerance in this study.

### CR-Induced Decrease in Plasma TG Is Abolished With PR

**Figure 8:**
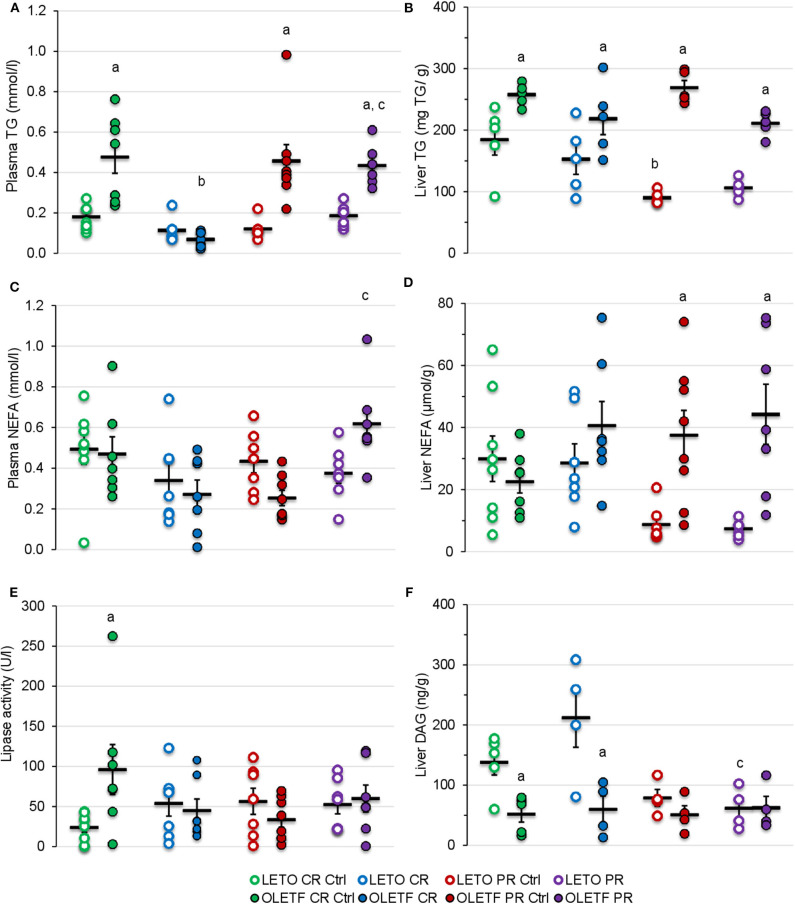
Mean ± SE **(A)** plasma TG (mmol/L), **(B)** liver TG (mg/g of tissue), **(C)** plasma NEFA (mmol/L), **(D)** liver NEFA (μmol/g of tissue), **(E)** plasma lipase activity (U/L), and **(F)** liver DAG (ng/g of tissue). ^a^*P* < 0.05 vs. LETO. ^b^*P* < 0.05 vs. Control. ^c^*P* < 0.05 vs. CR.

Mean plasma TGs were nearly 2-fold (*P* < 0.05) greater in OLETF compared to LETO at baseline, and levels decreased (*P* < 0.001) 86% in OLETF with CR compared to control (Figure 8A). After PR, levels in OLETF returned to baseline concentrations (*P* = 0.941; Figure 8A). Liver TGs were higher in OLETF compared to LETO throughout the study, but hepatic levels were not altered by either treatment in OLETF (Figure 8B). In LETO, liver TGs were lower (*P* = 0.002) in PR controls than CR controls, which maybe a reflection of a time (age effect), and levels were not significantly altered with either treatment (Figure 8B).

Plasma NEFA concentration were higher in OLETF following PR compared to the levels following CR (*P* = 0.002; Figure 8C). Otherwise, plasma NEFA levels remained constant in LETO regardless of treatment. CR did not induce a significant effect in either strain on hepatic NEFA concentrations, but because levels remained constant in OLETF and decreased in LETO, a strain effect (*P* < 0.05) was detected during the PR phase (Figure 8D).

Mean plasma lipase activity was nearly 3-fold higher (*P* = 0.003) in OLETF at baseline, but otherwise levels were stable within strain and were not altered by treatment (Figure 8E).

CR did not alter mean hepatic diacylglycerol (DAG) content in either strain, but levels remained higher (*P* = 0.009) in LETO than OLETF (Figure 8F). PR had no effect on levels in in either strain, but were reduced (*P* < 0.05) in both control and PR LETO compared to values following CR (Figure 8F). The lack of DAG accumulation in liver, particularly in OLETF after CR, suggests that lipolysis of TGs remained static. This is particularly beneficial as hepatic DAG accumulation is associated with increased insulin resistance more so than NEFA accumulation (37, 38).

### CR Increased Relative Plasma Creatinine in LETO, but Not in OLETF, While It Decreased Urea in OLETF

Mean relative plasma creatinine in LETO increased 75% (*P* < 0.001) after CR compared to control, but the effect of PR was not significant. Treatment effects in OLETF were not detected for creatinine, but plasma urea decreased 46% (*P* < 0.001), and by consequence urea: creatinine ratio (49%, *P* = 0.016), after CR, returning to basal levels after PR compared to PR control. Mean relative plasma urea did not exhibit a treatment effect in LETO (Table 2). We hypothesized that the transient decrease urea in the OLETF without increase in creatinine concentration can be attributed to protein depletion (39) rather than diabetic nephropathy, as the latter develops later in the OLETF (34).

## Discussion

Caloric restriction ameliorates metabolic syndrome in obese individuals (40), even in the presence of T2DM (13, 41). However, only a few studies have demonstrated the consequences of interrupting caloric restriction (42) without providing sufficient insight on the molecular mechanisms involved. The aim of this study was to provide further insights into how CR ameliorates metabolic syndrome, and how recovery of BM due to CR interruption abolishes these benefits.

### Caloric Restriction Promotes Loss of Lean Tissue and Water Rather Than Adipose

BM gain and food intake were consistently higher in OLETF compared to LETO, which is consistent for this model of diet-induced obesity (24, 25). The acute phase of severe CR resulted in nearly a 30% greater rate of mass loss in OLETF compared to LETO yet the loss of epididymal and retroperitoneal mass accounted for only about 5% of the total mass loss in OLETF suggesting that there was significant loss of lean tissue and the associated water. The loss of the primary adipose depots in the relatively lean LETO accounted for closer to 10% of the total BM loss suggesting that acute, severe CR in the healthier strain was less detrimental with respect to preserving lean tissue and total body water. Thus, these data suggest that during a diabetic condition that presents with metabolic syndrome, acute, severe CR may result in a disproportionate loss of lean tissue and water over the desired effect of preferential loss of excess fat mass (43). The loss of intracellular water derived from muscle catabolism may be a significant contribution to the total BM loss as has been observed in malnourished populations (44). In perspective, an extended bout of CR during a condition of metabolic syndrome has the potential to induce more severe cachexia and loss of total body water although the benefit of improved glucose tolerance could still be present. Thus, an appropriate balance between benefits in glucose tolerance and perseverance of lean tissue would need to be struck if this degree of CR was to be implemented as a treatment modality.

For both strains, the rate of mass recovery was greater than the normal growth rate for *ad libitum* control rats suggesting that the CR animals compensated for the rapid loss of mass by disproportionately increasing their food intake during the PR phase. This PR phase may have been associated with a decrease in basal metabolism to facilitate the rapid mass gain (45), consistent with lower BM (i.e., lower caloric balance) without obvious change in physical activity, which further widens the energy gap (energy intake vs. expenditure) (46). Interestingly, although mass loss and regain trends were similar for both strains, only OLETF rats had significant fat mass loss, which was not completely recovered after the PR phase. These observations suggest that the recovery of BM, most likely lean tissue, is prioritized in diabetic OLETF animals, more so than in lean, LETO.

### Caloric Restriction Did Not Ameliorate the Elevated SBP Despite Reductions in Adipose Mass

Differences in SBP between strains were detected 2 days after starting the caloric restriction (at 15 weeks) consistent with the hypertension in OLETF at 14 weeks (47). Obesity, especially visceral adiposity, is a known independent risk factor for hypertension, although several studies suggest that insulin sensitization induced by CR are also associated with a decrease in SBP (48). Even a modest decrease in BM (5–10%) can normalize blood pressure in obese patients (10). Metformin-induced increase in glucose tolerance elicited a BM-independent reduction in SBP of more than 10 mmHg in hypertensive OLETF rats (49) suggesting that an improvement in glucose tolerance may alter arterial pressure independent of reducing BM. Despite the profound reductions in adiposity and improvements in glucose tolerance in OLETF, these benefits did not translate into sustained or modest reductions in SBP. However, the reductions in SBP with metformin not observed here with CR may reflect off-target effects of metformin (i.e., pharmaceutical vs. behavioral interventions). Paradoxically, CR increased SBP in LETOs, which was normalized to control LETO levels after partial mass recovery suggesting that during non-diabetic conditions, potential stress, independent of the renin-angiotensin-aldosterone system (RAAS), during CR may have been sufficient to induce an increase in SBP.

The exact mechanisms promoting the strain-associated hypertension are not well-defined in OLETF; however, elevated RAAS is a likely contributing factor (26, 28, 50). The nearly 2-fold greater plasma aldosterone levels at baseline in OLETF compared to LETO substantiate the previous studies that increased RAAS is a contributing factor in the strain-associated hypertension. However, in the present study, the substantial reductions in BM with CR and the reciprocal increases in BM in the regain (PR) phase did not alter SBP or plasma aldosterone despite improvements in glucose tolerance suggesting that the benefits of acute, severe CR do not translate into amelioration of the hypertension and that the elevated RAAS is resistant to changes in BM and/or adiposity.

### SGLT2 Expression Is Sensitive to Perturbations During Metabolic Syndrome

In order to assess the contributions of renal glucose absorption via SGLT2, its expression was measured. The lack of changes in SGLT2 expression in LETO regardless of perturbation suggests that renal glucose handling in healthy animals is constant and robustly regulated. However, the changes in expression in OLETF following the perturbations suggest that the regulation of SGLT2 expression is more sensitive to variable aspects associated with mass loss and regain during diabetic conditions. Nonetheless, these differences in expression were not significant nor translate into robust biological effects.

Aside from the potential contributions of renal glucose handling, we can't discount the potential contributions of intestinal reabsorption on improving glucose tolerance. Unfortunately, we were not able to measure SLGT1 and GLUT2, the primary monosaccharide transporters in the intestine. However, these transporters are upregulated after feeding, especially after a glucose challenge (51). Therefore, we suspect they would be downregulated with CR and, at least, partially rebound during the regain phase.

### Despite Basal Differences in Gluconeogenic Enzymes, CR, and PR Did Not Induce Changes in Gluconeogenesis

Binding of insulin to its hepatic receptor activates a signaling cascade that inhibits the expression of the gluconeogenic enzymes, G6Pc, and PEPCK, in favor of increasing glucokinase expression and hepatic sequestration of glucose (52). However, the increased expression of the basal levels of both enzymes in the OLETF compared to LETO rats in the presence of similar insulin levels for both strains suggests that the liver is resistant to insulin. Moreover, the increased levels of basal glucose in the OLETF rats without significant changes in the potential for increased kidney glucose reabsorption (assessed via SGLT2) suggests that hepatic glucose production could be a major contributor in the systemic hyperglycemia during diabetic conditions that is not profoundly altered with CR nor PR. This is especially alarming if severe CR fails to suppress the relatively elevated expression of hepatic gluconeogenesis, even in the presence of a potential increase in insulin-stimulated glucose uptake in muscle via Akt2 activation after acute (53) and prolonged (54) CR. The combination of these factors could impair further potential benefits of CR, reflected by an improvement in IRI in the present study.

### Increased Adiponectin May Contribute to Amelioration of Peripheral IR

CR did not statistically alter adiponectin, an insulin-sensitizing adipokine (55), but levels increased after partial recovery in both strains. Moreover, leptin and leptin: adiponectin ratio decreased after CR, while leptin remained below basal levels after PR. These data suggest that the modest increase in adiponectin, however statistically insignificant, in the presence of suppressed leptin, reflected in the decreased leptin: adiponectin ratio, may be a more critical marker of the potential insulin sensitizing phenomenon in peripheral tissues than the changes in adiponectin levels alone following a bout of CR. Moreover, the decrease in leptin paired with loss of adipose tissue after CR in OLETF suggests that the hyperleptinemia present in the OLETF is a consequence of greater adipose mass rather than a derangement in leptin sensitivity and/or secretion (56). The increase in adiponectin following PR may be a compensatory response to protect against leptin-induced inflammation (19). Interestingly, this increase in adiponectin following PR was not sufficient to maintain the improvements in glucose tolerance because only partial recovery of the lost BM induced by CR was enough to abolish them. Thus, other aspects associated with the partial recovery of BM, which was not primarily adipose, contributed to the reversal of the CR-induced benefits on glucose tolerance.

### Few Metabolic Improvements Are Maintained After Only Partial Recovery of BM in a Model of Metabolic Syndrome

Acute CR improved glucose tolerance and reduced IRI in a model of metabolic syndrome likely through a reduction in hepatic gluconeogenesis, increased peripheral tissue glucose utilization induced via enhanced insulin sensitization, and increased NEFA uptake most likely by muscle. These improvements were accomplished despite minimal reductions in intraperitoneal adipose mass and significant reductions in lean tissue. Upon partial recovery of BM, the only metabolic change of note that we observed with some potential benefit was increased adiponectin; however, this change was not sufficient to maintain the benefit in glucose tolerance and reduced IRI despite only modest recovery in adipose mass suggesting that the very modest changes in adipose mass likely had minimal effects on the observed metabolic alterations.

## Data Availability Statement

The datasets generated for this study are available on request to the corresponding author.

## Ethics Statement

This animal study was reviewed and approved by the institutional animal care and use committee of Kagawa Medical University (Kagawa, Japan).

## Author Contributions

MC, AN, DN, and RO conceived and designed research. MC, JN, JC, and BE performed experiments and analyzed data. MC, JN, JC, BE, AN, DN, and RO interpreted results of experiments and approved final version of manuscript. MC prepared figures and drafted manuscript. RO edited and revised manuscript.

## Conflict of Interest

The authors declare that the research was conducted in the absence of any commercial or financial relationships that could be construed as a potential conflict of interest.
